# Carcinoma Ex Ameloblastoma of the Mandible: A Rare Case Report

**DOI:** 10.7759/cureus.49536

**Published:** 2023-11-27

**Authors:** Prachurya Dakshinakabat, Abikshyeet Panda, Pallavi Mishra, Monalisha Mahapatra, Lipsa Bhuyan

**Affiliations:** 1 Department of Oral Pathology, Kalinga Institute of Dental Sciences, Kalinga Institute of Industrial Technology (Deemed to be University), Bhubaneswar, IND; 2 Department of Oral and Maxillofacial Pathology, Kalinga Institute of Dental Sciences, Kalinga Institute of Industrial Technology (Deemed to be University), Bhubaneswar, IND

**Keywords:** secondary ameloblastic carcinoma, malignant ameloblastoma, ameloblastoma, carcinoma ex ameloblastoma, ameloblastic carcinoma

## Abstract

Ameloblastoma is a benign yet locally aggressive tumor of the jaw bones and is most commonly found in the lower mandibular region. Histologically, it shows benign characteristics. However, ameloblastoma can turn malignant to show a more aggressive clinical course. Carcinoma ex ameloblastoma is an extremely rare malignancy arising from a pre-existing long-standing ameloblastoma or a recurrence of an ameloblastoma. According to the literature search, six to seven cases have so far been documented, and the majority of the lesions had a propensity to metastasize. Here, we present a case of carcinoma ex ameloblastoma implicating a 19-year-old male patient manifesting in the mandible, which arises from pre-existing ameloblastoma.

## Introduction

The World Health Organization (WHO)’s classification system for odontogenic tumors divides malignant ameloblastic lesions into malignant (metastasizing) ameloblastoma and ameloblastic carcinoma (AC) [1-6]. The WHO (2022 edition) later divided them into AC de novo (primary type) and carcinoma ex ameloblastoma (secondary type) [7,8]. The secondary type of AC is further subdivided into two subtypes: The peripheral form of AC develops within a benign peripheral ameloblastoma, whereas the intraosseous type of AC occurs within a pre-existing benign intraosseous ameloblastoma [9-12]. Carcinoma ex ameloblastoma is extremely rare, and so far, few cases have been reported. Most of the patients were presented with fast-growing swelling in the lower region of the mandible, and very few cases were presented in the maxilla. The histopathological features of carcinoma ex ameloblastoma showed the combining features of ameloblastoma and cellular atypia [2,5,13]. Here, we report a case of carcinoma ex ameloblastoma affecting a 19-year-old male patient, with a detailed medical history, radiographic findings, and histopathological characteristics that supported the definitive diagnosis of carcinoma ex ameloblastoma.

## Case presentation

**Figure 1:**
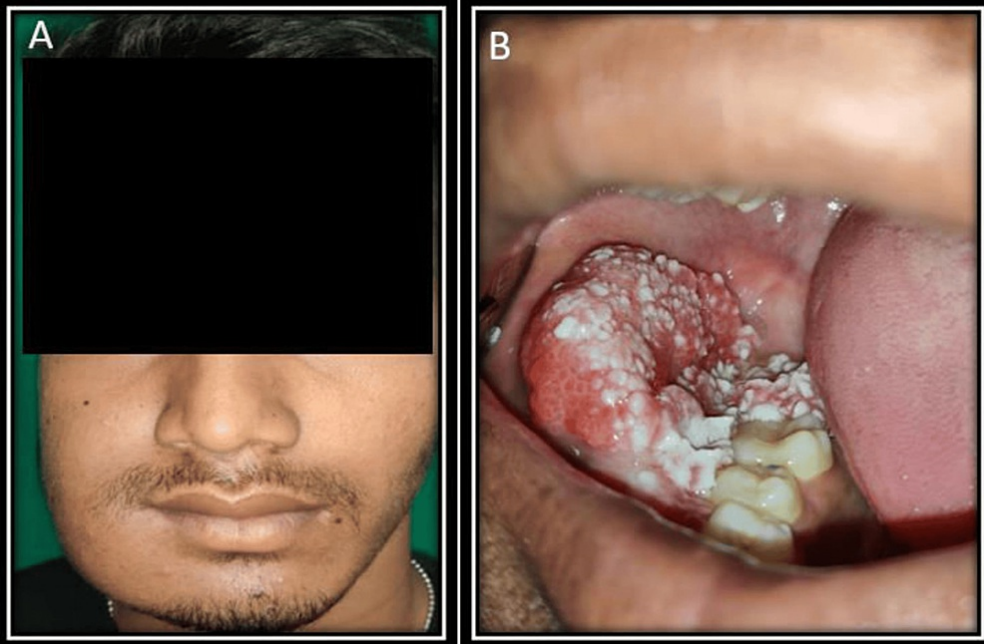
Clinical presentation of the patient. A: Extraoral image showing swelling in the right side of the face. B: Intraoral image showing an ulceroproliferative growth in the right side of the posterior mandible.

A 19-year-old male patient came to the outpatient department of Kalinga Institute of Dental Sciences, Bhubaneswar, India, with the chief complaint of painful swelling on the right side of his face for the past five years, which gradually increased. The patient had a habit of smoking for the last five years, which was continuing till he was admitted for the treatment. Extraorally, there was a diffuse firm swelling, which was tender on palpation, extending from the para-symphysis to posterior border of the mandible antero-posteriorly and from the line joining the right commissure of the mouth to the tragus to the lower border of the mandible supero-inferiorly resulting in evident facial asymmetry (Figure 1A). Intraorally, there was a diffuse swelling extending from the 43 region to the 48 region invading the retromolar area with vestibular obliteration. There was a history of pus discharge from the first and second molar region along with vestibule obliteration (Figure 1B). The swelling had ulcero-proliferative growth on its surface. The patient was unable to occlude due to the hindrance produced by the ulcero-proliferative growth on the occlusal surface of the posterior mandibular molars. The mouth opening was restricted to 25-30 mm. There was no difficulty in opening the jaw and no deviation evident.

**Figure 2:**
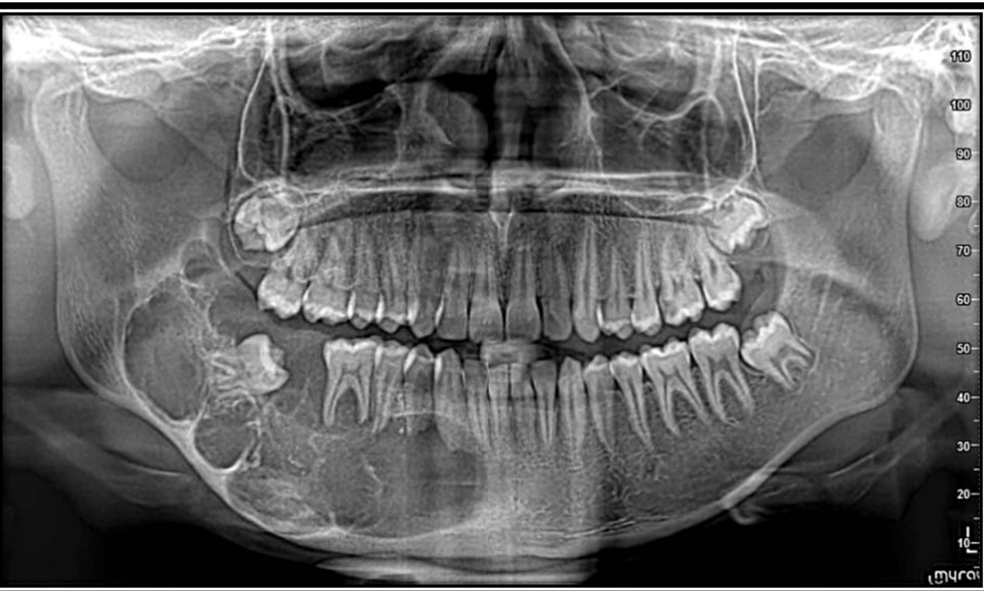
Orthopantomograph (OPG) image showing well-defined multilocular radiolucency extending from the distal side of 42 till the ascending ramus of the right side of the mandible.

Orthopantogram (OPG) revealed a multilocular radiolucency extending from the distal aspect of the root apex of 42 to the ramus of the mandible. The radiolucency had a soap bubble appearance with lower cortical expansion. There was evidence of root resorption related to 44, 45, and 46 (Figure 2).

**Figure 3:**
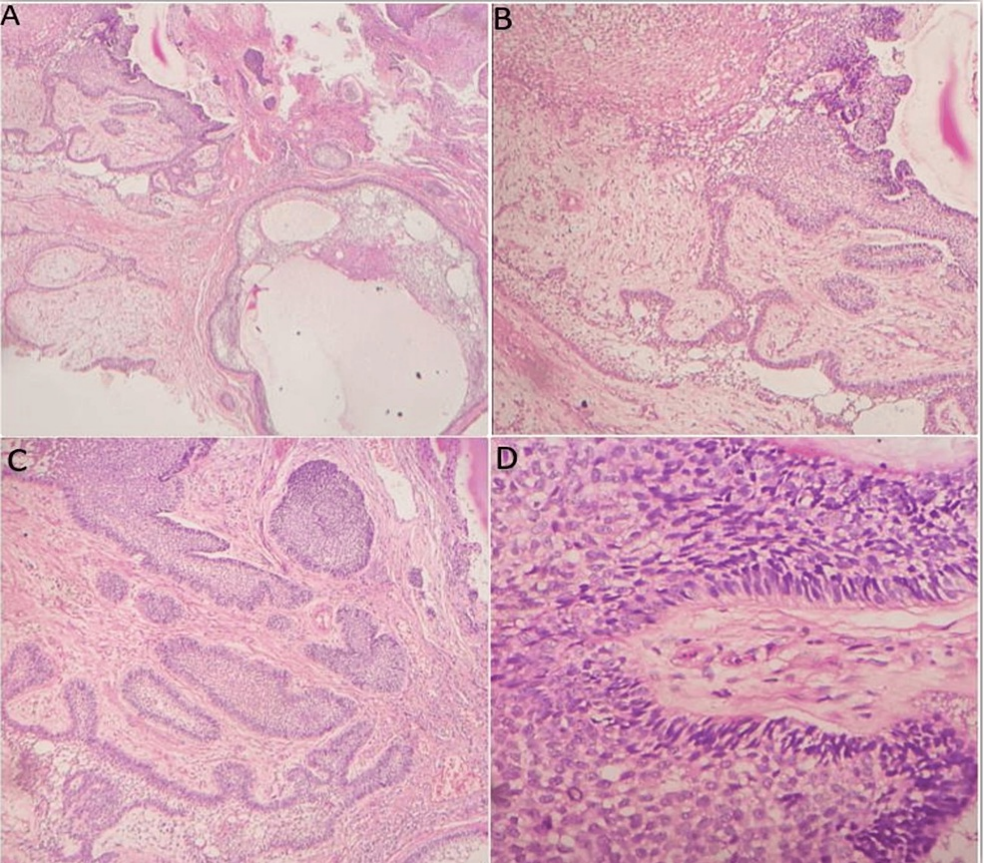
Histopathological section of the hematoxylin and eosin (H & E) section. A: 10x image showing multiple islands of ameloblastic epithelium in the fibrous connective tissue stroma. B: 10x image showing the epithelial island in the follicular pattern of the arrangement along with the central stellate reticulum. C: 10x image showing ameloblastic epithelial cells lost the central stellate reticular pattern and progressing toward the hypercellularity nature. D: 40x image showing that peripheral tumor cells are columnar along with reverse polarity and nuclear palisading, whereas the centrally placed tumor cells are showing hyperchromatic nuclei, multiple nucleoli, and abnormal mitotic figures.

An incisional biopsy was sent for histopathological examination. On gross examination, the incised specimen was whitish in color and firm in consistency. The hematoxylin and eosin-stained sections revealed a dense fibro-cellular connective tissue stroma covered by para-keratinized stratified squamous epithelium. The odontogenic epithelial tumor cells were arranged in follicle patterns. Individual tumor islands or cords consisted of two cellular features. The peripheral cells were columnar cells with reversal of polarity and nuclear palisading with centrally located loosely arranged polyhedral angular cells resembling the stellate reticulum. Few of the follicles are highly cellular showing hyperchromatic nuclei and nuclear atypia along with the squamous metaplasia features. A few follicles were merged to the surface epithelium where the maximum amount of atypia and a few abnormal mitotic figures are noticed (Figure 3).

The patient had undergone hemimandibulectomy along with radial neck dissection, and the biopsy specimen was sent to the lab for confirmatory diagnosis. The report was identical to the initial report, and the final diagnosis of carcinoma ex ameloblastoma was confirmed.

## Discussion

We discuss a case of AC that arose in the mandible of a 19-year-old male patient. Based on the clinical and histopathological examination, the case was diagnosed as carcinoma ex ameloblastoma (secondary type-dedifferentiated). Only 37 cases were reported till the year 1984 in the literature, while 138 cases of AC were reported in the literature till the year 2018 [3-6]. Most of the cases occurred as the primary type and were reported in the mandible. To date, few cases of malignant transformation from ameloblastoma (secondary type) have been documented, most of which were reported in the mandible. Thus far, only two to three cases have been reported in the maxilla [4,6,7], five to six cases of which transformed into carcinoma after their recurrence [2-8]. Only six to seven cases were reported, where carcinoma was developed from pre-existing ameloblastoma, and here, we compare the histopathology of our case with previously reported cases.

Typically, malignant cytological characteristics combined with the histological pattern of an ameloblastoma characterize the histopathological features of AC. A tall columnar cellular morphology and peripheral palisading with reverse nuclear polarity-type cell morphology are usually found. There is evidence of a central stellate reticulum-like structure in most of the previously reported cases. Along with pleomorphism, mitotic activity and multiple nucleoli are the prominent features [8,12,13]. The present case exhibited malignant features histopathologically without metastasis, leading to a diagnosis of carcinoma ex ameloblastoma, which arose from pre-existing ameloblastoma. The histopathology showed features of a follicular ameloblastoma along with neoplastic changes, such as nuclear hyperchromatism, aberrant mitotic figures, altered nuclear-cytoplasmic ratio, and multiple nucleoli. The present case also showed squamous metaplasia with proliferative activity in numerous follicles. Few follicles even assumed the morphology of sheets of neoplastic cells. These neoplastic cells demonstrated features of atypical cells of epidermoid carcinoma This is a unique feature scarcely reported in the literature.

Ameloblastoma has been histologically thought of as a benign tumor that has the potential to be locally aggressive, and malignant change from the benign tumor is not uncommon in locations other than the lower mandible. When ameloblastoma is detected, carcinoma should always be taken into consideration [5].

According to Akrish et al., the combination of histologic changes with demographic characteristics and biological behavior is necessary for the differential diagnosis between ameloblastoma and carcinoma ex ameloblastoma [5]. There should be the following histopathological changes: 1) An increased proliferative index is emphasized by increased mitotic activity, increased expression of the proliferating cell nuclear antigen, and increased Ki-67; 2) cellular atypia, such as nuclear pleomorphism and basilar hyperplasia; 3) hyperchromatic nuclei of basaloid cells; and 4) additional malignancy-related characteristics, such as perineural or perivascular invasion. This needs to be compared to the clinical and biological characteristics.

Based on the literature search, only seven articles were found on “carcinoma ex ameloblastoma” that match the features of our case [2,3,4,5,6,7,8] (Table 1).

**Table 1:** Review of previous reported cases of carcinoma ex ameloblastoma

Author name, year	Age	Gender	Location	Recurrence
Cox et al., 2000 [2]	25 years	M	Mandibular molars	Yes
Cizymecy et al., 2004 [3]	44 years	F	Mandibular molars	Yes
Akrish et al., 2007 [4]	80 years	M	Anterior and posterior mandible	Yes
Abiko et al., 2007 [5]	72 years	M	Anterior and posterior mandible	N/A
Lin et al., 2013 [6]	30 years	M	Anterior maxilla	Yes
Kosanwat et al., 2018 [7]	46 years	F	Anterior maxilla	Yes
Ngokwe et al., 2023 [8]	64 years	F	Anterior Maxilla	Yes

In all the previously diagnosed cases, the patients were reported with the expansion of the mandible, severe bone loss, pain, and paraesthesia after eight to 10 months due to denial of the treatment. Here, in the present case, the patient reported an extra-oral swelling, which was rapidly growing in nature, and an intraoral ulceroproliferative growth in the lower molar region. The patient had a habit of smoking for the past five years, which could be the predisposing factor for the malignant transformation. Based on the clinical and histopathological features, the case was diagnosed as carcinoma ex ameloblastoma. Initially, the patient denied the treatment after he received the incisional biopsy report. Later on, he reported back to the department with severe pain and swelling and underwent hemimandibulectomy with radical neck dissection. The excisional specimen concurred with the incisional report.

## Conclusions

Carcinoma ex ameloblastoma is a rare entity when compared to AC and malignant ameloblastoma. Therefore, proper clinical evaluation along with histopathological examinations will be helpful for diagnosis and treatment planning. Histopathological examinations of carcinoma ex ameloblastoma are quite difficult because it exhibits a variety of microscopic features, so its diagnosis should be taken into consideration due to its rarity. The primary cause of concern is that recurrence was observed in nearly all of the prior cases. Hence, long-term follow-up and health screening are necessary for every reported case.
