# Correction: Carcinoma Ex Ameloblastoma of the Mandible: A Rare Case Report

**DOI:** 10.7759/cureus.c168

**Published:** 2024-03-25

**Authors:** Prachurya Dakshinakabat, Abikshyeet Panda, Pallavi Mishra, Monalisha Mahapatra, Lipsa Bhuyan

**Affiliations:** 1 Department of Oral Pathology, Kalinga Institute of Dental Sciences, Kalinga Institute of Industrial Technology (Deemed to be University), Bhubaneswar, IND; 2 Department of Oral and Maxillofacial Pathology, Kalinga Institute of Dental Sciences, Kalinga Institute of Industrial Technology (Deemed to be University), Bhubaneswar, IND

This article has been corrected to replace an incorrect reference. The original published article incorrectly cited the following article as reference 8:

Hayashi N, Iwata J, Masaoka N, Ueno H, Ohtsuki Y, Moriki T: https://dx.doi.org/10.1007/s004280050061 Ameloblastoma of the mandible metastasizing to the orbit with malignant transformation. A histopathological and immunohistochemical study. Virchows Arch. 1997, 430:501-7. https://dx.doi.org/10.1007/s004280050061 10.1007/s004280050061

Reference 8 has been updated with the correct article:

Zilefac BN, Ntep B, Bola S, Kamdem SN, Zacharie S: https://dx.doi.org/10.1016/j.adoms.2023.100413 Secondary maxillary ameloblastic carcinoma: A case report. Adv in Oral and Maxillofac Surg. 2023, 10:100413. https://dx.doi.org/10.1016/j.adoms.2023.100413 10.1016/j.adoms.2023.100413

